# Supplementary material for: Re-assessing the role of culture on the visual orientation perception of the rod and frame test
Source: PLoS One. 2022 Oct 20;17(10):e0276393. doi: 10.1371/journal.pone.0276393 (PMC9584537; doi:10.1371/journal.pone.0276393)
Supplement: Attachment — Submitted filename: Response to Reviewers.docx [file pone.0276393.s001.docx]

Greetings Dr. Alastair Smith,

We thank you and the two reviewers for your time with our manuscript. We appreciate the feedback, comments and suggestions. Please find our responses to the points made by the reviewers below.

Thank you,

Chéla Willey & Zili Liu

Reviewer #1: This study addressed cross-cultural differences in perception, specifically in visual perception, by using a visuospatial task that assesses the person's ability to align a rod to true vertical (gravity) or identify the direction of the rod when surrounded by a distracting tilted frame. The manuscript is generally well written, but there are a few concerns.

1. The authors can still enrich their Introduction with information on how culture influences attentional processes (direct attention to focal objects or contexts), and on the type of processing (analytical or holistic) different cultures prefer.

Thanks. We have now provided additional references in the introduction to the attentional processes and analytical vs. holistic account, as follows (lines 50-58).

“For example, when viewing photographs of natural scenes, American participants were found to fixate on the focal object more quickly and more often than their Chinese counterparts, who made more saccades to the background [3]. The theory behind such findings is the claim that Westerners tend to perceive analytically and independently of context, whereas Easteners perceive holistically and take context into consideration [4]. Such a claim basically predicts that Westerners are less vulnerable than their Eastern counterparts to visual illusions, since a large number of visual illusions are context-dependent.”

2. In the Results section, when referring to "Others", it would be useful to include what is meant by that. It is not certain whether the authors included this information in the included appendix of the questionnaire.

Agreed. We have now included additional information regarding the “Others” in the main text of the manuscript as follows:

For the alignment task (Lines 186-189):

“The remaining 135 participants were categorized as “Other”. Within the “Other” category, 38.5% identified as Hispanic/Latinx, 23.7% as Middle Eastern (including Persian, Armenian, Pakistani, and Israeli), 13.3% as Native American, 3.7% as African American, and 20.7% as multiracial. Within this “Other” category, 86 (64%) were female.”

and for the Discrimination Task (Lines 236-240):

“The remaining 79 participants were categorized as “Other” (61% were female). Within the Other category, 34.2% identified as Hispanic/Latinx, 27.8% as Middle Eastern, 7.6% as Native American, 7.6% as African American, and 22.8% as multiracial. Within this “Other” category, 48 (61%) were female.”

3. Also for "Others", it would make it easier for the reader if the gender distribution is specified for this group as well at each analysis trial.

This information has been included - see above for included text.

4. For generational data, it is not clear how long the 1st generation Americans or the non-US citizens have been in the USA. It is possible that the time of their stay may influence the extent to which they were affected by the culture they are exposed to.

Based on the definition in the survey question, 1st generation U.S. citizens were participants who were born in America. However, regardless if the participant was born in the U.S. or not, we do not know how long they lived in the U.S because we did not collect such data, unfortunately. We added a statement about this limitation in the discussion:

“One limitation of the current study is that length of stay in the U.S. was not collected from those who were non-citizens. As mentioned in the introduction, the effect of culture may be lessened between Caucasians and East Asian participants based on the length of stay and the immersion of East Asian participants in the westernized culture of the U.S. In the current study, given the sample size, even if the effect was lessened, statistical power was sufficient to detect even small differences between the cultures. Additionally, the small effect that we did observe was opposite of what has been discovered previously, suggesting more research should be conducted in this area.”

5. Line 223: it is not clear what is meant by " and interestingly since it was in the "wrong" direction of ethnicity".

We added further clarification in now line 246-248:

“...and of ethnicity, *F* (1, 137) = 4.76, *p* = .031, $\eta_{p}^{2}$ = .03, such that females and Caucasians showed greater biases.”

6. Lines 239, 240. This is a run-on sentence.

We fixed this sentence in the manuscript in line 262:

“We again found a main effect of sex, *F* (1, 218) = 12.39, *p* = .001, $\eta_{p}^{2}$ = .05, and an interaction of sex and frame tilt, *F* (1, 218) = 15.44, *p* < .001, $\eta_{p}^{2}$ = .07.”

7. The figures are not numbered. They should be.

The submitted figure file names were numbered per the instructions of the journal and the figure titles in-text were also numbered to correspond with these file names. We aren’t sure how to edit this for further clarification.

8. In the figure on generation effects (I assume it is Figure 8), the authors should include number of subjects (n).

Thank you. Added sample sizes to Fig 8.

9. It would be worth adding how sex hormones influence performance on visuospatial tasks either in the Introduction or Discussion to delineate any gender effects.

Thank you for the suggestion, we added this in lines 400-407:

“We found that females showed a larger bias than males in judging the rod’s vertical orientation, when the frame was tilted. This finding was consistent with previous findings in the literature [12]. However, given that sex difference was not a main focus of the current study, we did not collect any menstrual phase information from our female participants to correlate between the size of the bias and menstrual phase (as a proxy of sex hormones), as did in [12]. As such, this finding in the current study served only as confirmation that the non-cultural aspects of our data were consistent with the literature, giving some credence to the quality of our data.”

Reviewer #2: This is a short and interesting study but some elements, which are overall minor in my opinion, should be improved before it can get published. Below are my point-by-point comments about the paper.

Line 83. Is there a justification for using a 3D version of the RFT here? Is it because it may increase the effect size and therefore be more sensitive to detect cultural variations?

Yes, the real life RFT and the 3D rendered RFT have shown greater biases toward the tilted frame than 2D paper and 2D computer rendered RFTs. The effect sizes in biases we have found in the current study are similar to what others have found in both studies using a real-life RFT and a 3D rendered RFT in virtual reality. Given the larger effect sizes, we reasoned that any cultural differences would be more apparent when using a 3D version. We opted for the virtual reality version due to our ability to better control for visual cues and to allow for the ability to perform the discrimination task (controlling for the amount of time participants are exposed to the stimuli).

Line 83. How is culture operationalised and tested? Is culture tested in different countries? (one discovers that this is not the case when reading the paper, but this should be prepared). Is there a justification/good reason for testing culture only within the US? One may refer to studies which found that cultural effects are smaller/decrease for Asians living in the US, or Amercians living in Asia (e.g., Kitayama,Duffy,Kawamura, and Larsen, 2003)

Great point. We have revised to mention this detail about our sample earlier in the paper and have added this finding in the introduction:

Line 57-58: Though these cultural effects may be lessened as participants of one culture have immersed themselves in the other culture [(5)].

Line 70: “The aim of the current research is to re-investigate cultural differences of those living in the United States (either in their life time or recent international arrivals) on the RFT, a task that measures the influence of a contextual frame on basic orientation perception”

Line 84. The meaning of "to anticipate" here is difficult to grasp. I had to read the sentence twice to understand its meaning. It was not clear to me, at first, that this was a preview of all the findings of the study.

Thank you, we have revised this for further clarification.

Line 92: “To preview, in the current study we tested and found the typical bias caused by a tilt of the frame in the RFT.”

Line 111. Lack of clarity, the sentence suggests that there were three conditions (-18°, 0°, 18°), with 6 trials each. Is this correct? If yes, could this be said explicitly?

Revised for clarity.

Line 118-119: “They completed six alignment estimates to vertical for each of three tilted frame conditions: -18°, 0°, +18°”

Line 158. A description of how culture is defined, in subsequent analyses, would be useful. In addition, given that culture is the main IV in this study, it is important to provide specific information about the questions asked to the participants in order to classify them.

Exact survey questions added. Additionally, the following was added to the text beginning on

Line 167:

“Important to the topic of this paper, participants were asked to self-identify their ethnicities as well their nationalities (which nation for which they claimed citizenship) with open ended questions. Two independent raters categorized participants’ ethnicity as Caucasian, East Asian, or Other based on their written responses. Those categorized as Caucasian included those who self-identified as “white”, “Caucasian”, and with other European ancestry. Those categorized as East Asian included those who self-identified as “Asian”, Chinese, Taiwanese, Japanese, Korean, and Vietnamese. All participants outside of these identifiers including those who were multiracial were categorized as “Other” for this investigation.”

Line 166. Who are the 137 participants who do not identify as EA or Caucasian? How is Caucasian actually defined? Does it mean white skin? What about participants of mixed origins, are they other? We are given little information about the main IV of the study, and this is problematic.

We added more information in the manuscript about the questions participants answered about their demographics and how they were subsequently categorized, see response above. Demographic breakdown of the “Other” category was added (see response below), which does include participants of mixed origins.

Participant responses to open-ended questions were also added to the data file linked in manuscript for further transparency.

Line 179. Does Ethnicity only have two levels? What about the 'other' group shown on the graphs? What does it mean exactly?

In the participants subsection of the method, we added information about the “Other” category with the breakdown of who is included in the sample.

In the alignment task:

“The remaining 135 participants were categorized as “Other”. Within the Other category, 38.5% identified as Hispanic/Latinx, 23.7% as Middle Eastern (including Persian, Armenian, Pakistani, and Israeli), 13.3% as Native American, 3.7% as African American, and 20.7% as multiracial. Within this “Other” category, 86 (64%) were female.”

and in the Discrimination Task:

“The remaining 79 participants were categorized as “Other” (61% were female). Within the Other category, 34.2% identified as Hispanic/Latinx, 27.8% as Middle Eastern, 7.6% as Native American, 7.6% as African American, and 22.8% as multiracial. Within this “Other” category, 48 (61%) were female.”

Line 190. Although the main effect of ethnicity is not significant, there may be a trend there. Same comment as below.

Line 195. The main effect of Frame suggests that overall performance varied across males and females. Were RTs measured, in ordre to check for possible engagement effects? (i.e., longer RTs, coupled with better overall performance and lower illusion size?)

Unfortunately, RTs were not measured in the current experiment. We agree that there should be a speed-accuracy trade-off though we did not anticipate that RT would covary with ethnicity or sex.

Line206. How is a second generaion Asian defined? Is it enough to have one (out of 4) grandparents of Asian decent to be considered a 2nd-generation Asian?

Generation is in reference to how many generations the participant’s family had been in America. For example, if one self-identified as Asian, 1^st^ generation American, this would suggest that they were of Asian descent and at least one of their parents was born in a country outside of the US. Thus, it was enough for one (out of 4) grandparents to be born in another country to be considered a 2^nd^ generation American. We included additional information about the generation survey question within the manuscript beginning in Line 177:

“First generation meant that the participant was a citizen, but was not born in the U.S. Second generation was defined as being born in the U.S., but at least one parent was born in another country. Third generation was defined as being born in the U.S., both parents were born in the U.S., but at least one grandparent was born in another country.”

Line 314. This conclusion is somewhat surprising. A number of analyses suggest that the performance of the two cultural groups differ. In fact, there is no strong evidence for an absence of difference. the fact that the difference does not go in the direction predicted by what is called the 'culture theory' (making it sound as though there is only one possible account for cultural effects), does not necessarily mean that there is no effect. Rather, it may mean that if the effect shown here actually exists (and one cannot really decide one way or another), it cannot be accounted for by the social organisation theory.

We agree with the reviewer that our overarching results cannot be accounted for by the theory put forward by Nisbett and colleagues that Easterners and Westerners differ in their basic visual perception of the RFT. We also agree with the reviewer that there is certainly more than one account for cultural effects, although we hope that the context made it unambiguous that we specifically referred to that proposed by Nisbett and colleagues. Nevertheless, the majority of our results found no differences between the two cultural groups mentioned above. While we did find a difference in the opposite direction in some of the analyses, these analyses were not in the majority and the effect size found was small. As such, we prefer to be more cautious than claiming that the effect in the opposite direction was the main conclusion, particularly since we do not have a theory as to why this should be expected.

Line 373. The discussion feels short, with no attempt to account for the cultural effect observed in the study, and little discussion about the gender effect observed.

By “the cultural effect observed in the study,” we believe that the reviewer meant the effect found that was in the opposite direction as predicted by Nisbett and colleagues. We agree with the reviewer we did not attempt to account for such an effect, primarily because we are not aware of any theory predicting such an effect. We also feel that it is premature for us to offer an account before reconciliation can be found between our current study and studies in the literature that did not find consistent results. We hope that the reviewer can understand our position that we do not want to claim that our data are more accurate than those in the literature. Rather, we are seeking a way to make sense of all the data found so far.

We did add some more discussion on the gender effect observed as per Reviewer 1 suggestions. We tested for this known effect in order to confirm that the non-cultural aspects of our data were consistent with the literature, to allow for credibility of the quality of our data.
