# Supplementary material for: Re-assessing the role of culture on the visual orientation perception of the rod and frame test
Source: PLoS One. 2022 Oct 20;17(10):e0276393. doi: 10.1371/journal.pone.0276393 (PMC9584537; doi:10.1371/journal.pone.0276393)
Supplement: Attachment — Submitted filename: Response to Reviewers_3.docx [file pone.0276393.s002.docx]

Reviewer #1: The authors have addressed my previous comments, but I have a few more for this revised manuscript.

Overall, I feel that cultural studies should include participants within their cultural habitat for comparisons sake, rather than including participants of different generations in a country different than their own. Having said that, this is no implication to the significance of this study.

1. In the updated abstract, please use "white Western Europeans", rather than "white" participants, because at this point of the manuscript, the term has not been defined yet.

Thanks, abstract was edited per the suggestion.

2. Page 5, lines 89-90: Please put the reference [9] after the 2D computerized test, not in line 90 citation [9, 10].

Fixed, reference moved per the suggestion.

3. I feel that the authors included too many comments and even reference citations in the Results sections (3.1.1 SV Bias; 3.2.3 Generational Data); it is better to just report the findings in the Results section, and then leave the explanations for the Discussion section.

We edited these sections to move some of the commentary from the Results section into the Discussion section, which helped to reduce redundancy.

4. Why is the number of participants for Generational Data different in 3.1.2 and 3.2.3?

Section 3.1 describes data from all the participants who completed the alignment rod and frame task. Section 3.2 describes the data from the subset of participants who also completed the rod and frame discrimination task.

5. The authors state that they cannot offer an explanation for their paradoxical findings which contradicted their hypothesis and those from a previous study [Reference 6] that reported that East Asians were more prone to larger errors on the RFT, in comparison to their Western counterparts. Unexpectedly, other results also indicate that later generation Americans, which are quite immersed in American culture, displayed greater biases than the 1st generation Americans or non-citizens.

Whether methodology differences might have affected the results, specifically the RFT protocol, is not clear. Unlike Reference 6, participants in this study stood on a foam block while aligning the rod to vertical. This might be worth looking into.

Thank you. We have revised our discussion per your suggestion, as follows (lines 425 – 437):

In our experiment, all participants were in control of the rotation in the alignment RFT. This should have produced the best possible performance of the white males. However, our data showed the opposite, that East Asian males performed slightly better than white males. Also, in our study, white participants got worse in their discrimination abilities across generations, while their Asian counterparts did not change. We do not have an explanation why our data contradicted theirs or, more generally, why we could not replicate their results. Apparently, our virtual reality experiment was different from theirs using a physical RFT apparatus. Our participants also stood on a foam mattress while performing the RFT. It is unclear whether this methodologic difference could be responsible for the different results. However, as noted above, we have replicated the frame effects similar in magnitude to those found in other studies using the physical apparatus and a 3D virtual RFT. Regardless, these methodological issues are worth looking into. But it seems fair to say that the earlier cultural effects may not be as robust as previously thought.

Reviewer #2: I find that the revised version of the manuscript adequately addresses all the issues raised during the review.
